# Exosomes from mesenchymal stem/stromal cells and their therapeutic role in osteoarthritic pain

**DOI:** 10.1002/jeo2.70825

**Published:** 2026-06-26

**Authors:** Dimitrios Kouroupis, Stanislava Jergova, Jacqueline Sagen, Thomas M. Best

**Affiliations:** ^1^ Department of Orthopedics, UHealth Sports Medicine Institute, Miller School of Medicine University of Miami Miami Florida USA; ^2^ Diabetes Research Institute, Cell Transplant Center, Miller School of Medicine University of Miami Miami Florida USA; ^3^ Miami Project to Cure Paralysis, Miller School of Medicine, University of Miami Miami Florida USA

**Keywords:** calcitonin gene‐related peptide, exosomes, mesenchymal stem/stromal cells, osteoarthritic pain, substance P

**Abbreviations**
AAV: adeno‐associated virus
aCGRP: CGRP antagonist
CGRP: calcitonin gene‐related peptide
EVs: extracellular vesicles
EXOs: exosomes
IFP: infrapatellar fat pad
MSC: mesenchymal stem/stromal cells
OA: osteoarthritis
SP: substance P

## OSTEOARTHRITIS (OA) PHENOTYPES AND JOINT PAIN

**Figure 1:**
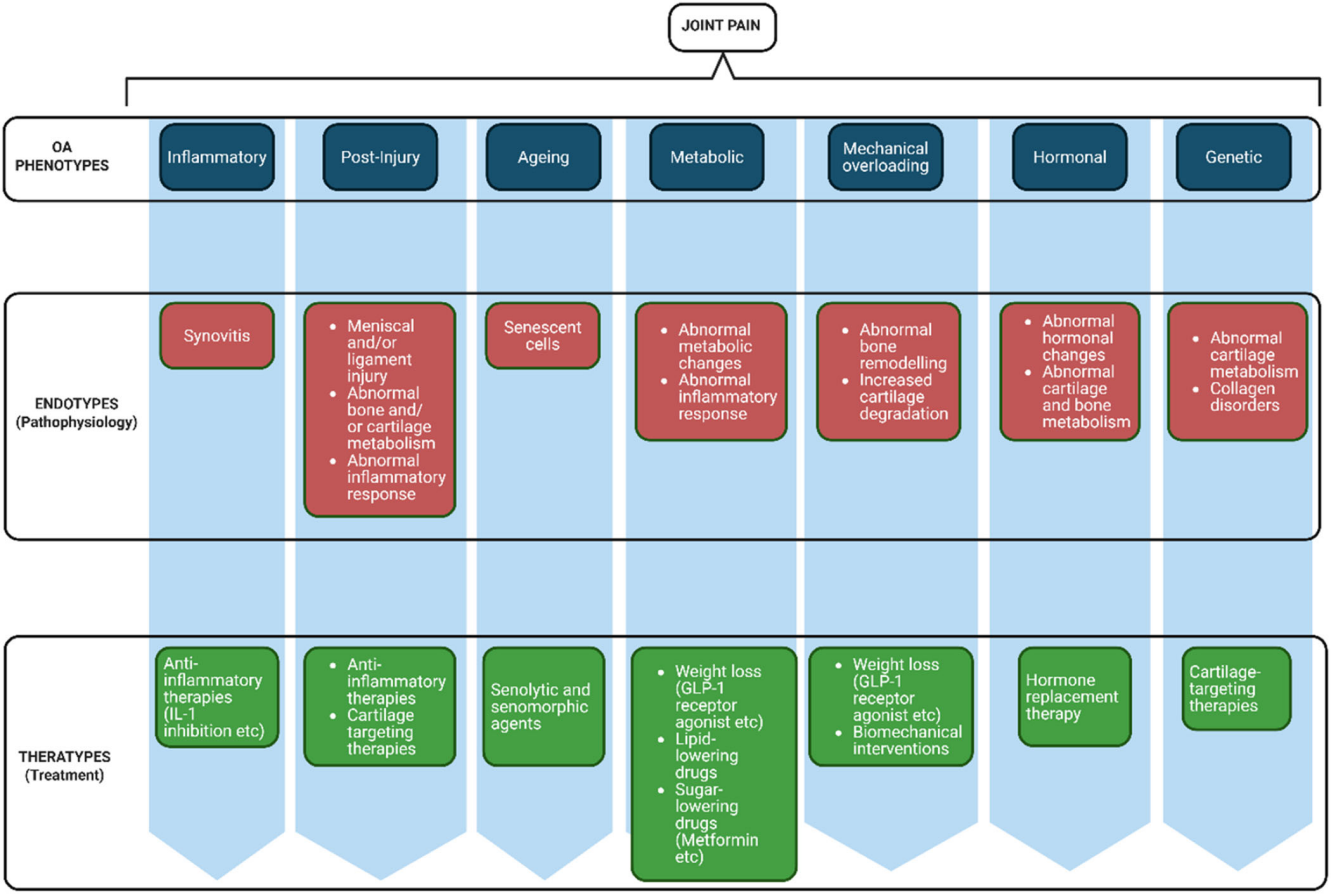
Proposed OA phenotypes all involving joint pain. Endotypes (pathophysiology) and theratypes (treatment) are directly related to the specific OA phenotype. OA, osteoarthritis.

Knee OA affects approximately 500 million people worldwide and is an increasingly common cause of disability and societal economic burden [78]. In the US alone, symptomatic knee OA affects approximately 14 million people, including 2 million under the age of 45 and another 6 million in the age range of 45–64 [20]. Collectively, OA ‘clinical phenotypes' [18, 19, 22, 24, 34] have similar clinical end‐points including pain, stiffness, joint damage and a common activation of local inflammatory/immune cascades [18, 19, 22, 34, 45] (Figure 1). Efforts continue to unveil novel therapies that will hopefully be effective to modify symptoms, importantly pain and loss of mobility and ideally the arrest of disease progression. Pain associated with OA is estimated to occur in approximately 10% of the global population over 60 years [81]. Relevant to this, the most common symptom of OA is joint and regional pain (https://www.niams.nih.gov/health-topics/osteoarthritis). Importantly, during disease progression, the synovium and infrapatellar fat pad (IFP) have become increasingly recognised as a site for immune cell infiltration, origin of pro‐inflammatory and AC degradative molecules, as well as a source of the pain‐transmitting, immune and inflammation modulator neuropeptides, Substance P (SP) and Calcitonin gene‐related peptide (CGRP) [6, 17].

### Joint pain signalling

Within the IFP, the neuropeptide SP is notable for its multiple effects on joint homeostasis, motivating us to revisit the activities of an ‘old’ molecule now in the context of early pathological events in conditions such as OA. In addition to established roles in nociception [41] (with its clinical implications), recent evidence supports SP participation in the modulation of local neurogenic inflammatory/immune responses [54, 76]. The proinflammatory effects of SP are mediated via the activation its functional receptors, the neurokinin 1 receptor (NK1R) receptor and mas‐related G protein‐coupled receptors X member 2 (MRGPRX2) [85]. SP increases vascular permeability favouring immune cell infiltration, while directly affecting macrophage phenotypic polarisation and migration to sites of inflammation [48, 50, 72]. Interestingly, studies showed that SP shows context‐dependent restorative and anti‐inflammatory effects related to dosage and type of receptors activated in vivo [43, 48, 84]. Therefore, despite the well‐described roles of SP in nociception and neurogenic inflammation, little is known about how to effectively inhibit SP signalling activity [57]. We believe that the resulting ‘neutralisation’ of pathologically‐expressed SP could have an impact on several key aspects of joint homeostasis (e.g., local immune responses), as well as joint pain and mobility.

**Figure 2:**
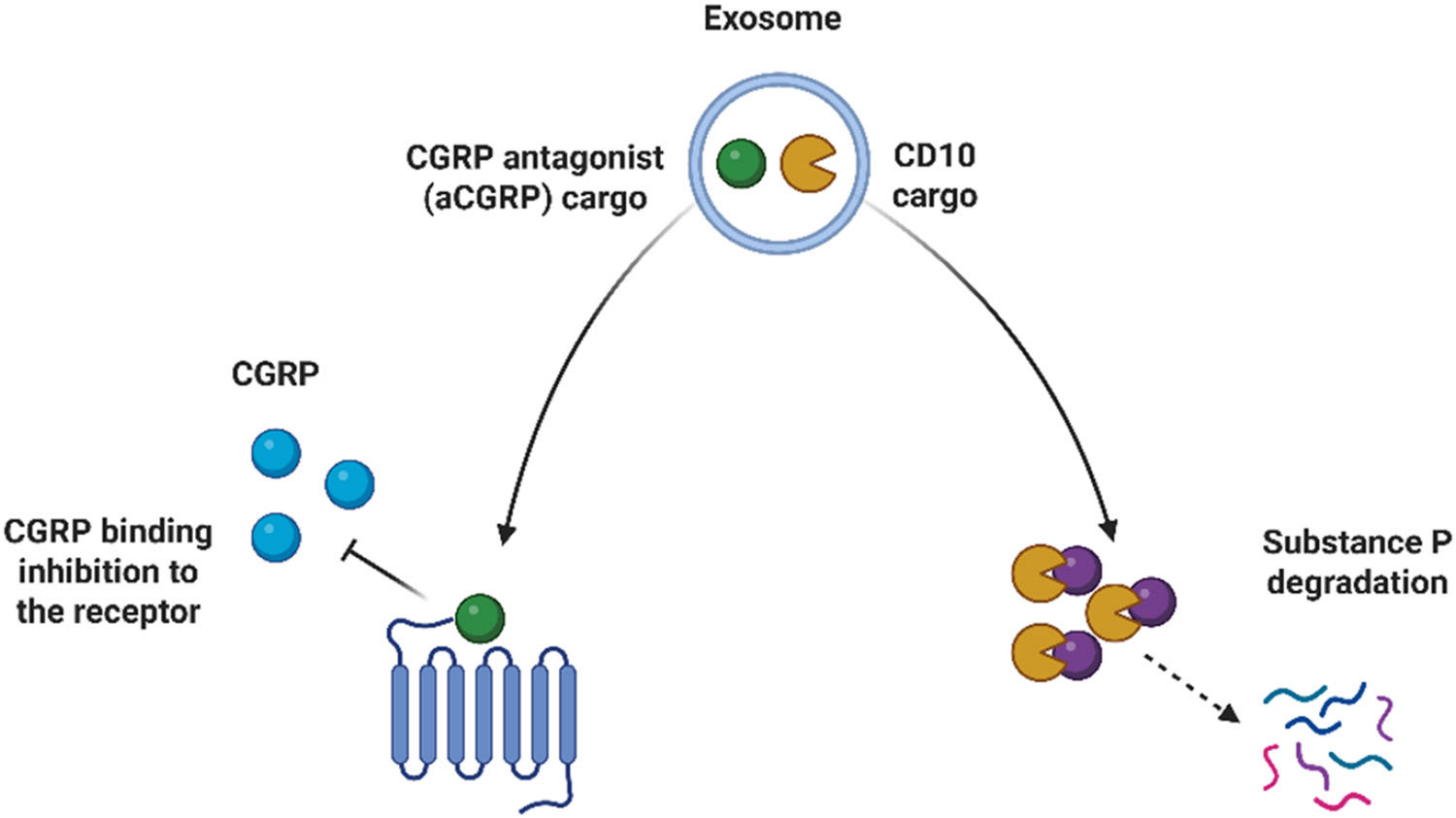
EXO product that uniformly exhibits a specific desired attribute CD10High (SP degradation and inflammation reversal) carrying pain‐reducing CGRP antagonist cargo (EXOs‐adeno‐associated virus [AAV] CGRP_8–37_). aCGRP, CGRP antagonist; CGRP, calcitonin gene‐related peptide; EXOs, exosomes; SP, substance P.

SP activity is regulated by the cell membrane‐bound (ectoenzyme) neutral endopeptidase CD10/neprilysin (reviewed in [53]), expressed in mesenchymal stem/stromal cells (MSC) [10, 11, 32, 59] and specifically in IFP‐MSC as we recently reported [39]. Based on its enzymatic activity, CD10 anti‐inflammatory effects have long been recognised in other systems [70, 71], while CD10 knockout mice have an increased propensity to inflammatory conditions such as colitis [73]. We recently reported significant CD10 enrichment in IFP‐MSC stimulated (i.e., primed) with inflammatory/pro‐fibrotic cocktails (tumor necrosis factor [TNFα]/interferon gamma [IFNγ]/connective tissue growth factor [CTGF]) or cultured regulatory‐compliant media, and effective SP degradation capacity by CD10High IFP‐MSC both in vitro and in vivo (Figure 2) [38, 39]. In addition, we consistently found a comparable SP degradative effect by the cells and their supernatant, abrogated by a CD10 inhibitor, suggesting an exosome‐mediated mechanism involving the release of CD10 from the cells. Exosomes (EXOs) are nanosized (50–200 nm) extracellular vesicles (EVs) generated via the endosomal pathway [73], and secreted by numerous cells in response to their surrounding milieu [83]. Harnessing these dynamic responses leads us to propose that we may customise their contents and identity (e.g., CD10^High^) by processing parental IFP‐MSC with specific protocols to produce enhanced effects. The potential therapeutic use of these cell‐free, EXOs‐based products has sparked multiple preclinical studies [5, 33, 56, 83], technical recommendations [61, 62, 64, 82] and clinical translation with encouraging initial results [36], rapidly motivating EXOs engineering for various clinical trials [33].

CGRP, a 37 amino‐acid peptide, is well documented in playing a key role in the transmission of pain signals in the periphery and CNS. Accumulating evidence implicates a role of CGRP in the peripheral mechanisms of OA pain [25, 27, 30, 65, 67]. Multiple knee joint structures are richly innervated by CGRP‐expressing sensory neurons [25, 68]. Synovial CGRP‐immunoreactive nerve fibre density is greater in OA patients with pain as compared to asymptomatic controls [67], and the pain localises to the joint compartment, exhibiting increased CGRP‐immunoreactive fibre density [13, 65]. In animal studies, intraarticular injection of CGRP caused mechanical allodynia in naïve mice, and CGRP receptor antagonist and CGRP neutralising antibody treatment alleviated OA‐associated pain [8]. Injection of Fluoro‐gold into the L5–L6 facet joint of rats caused an increase in CGRP in L1 and L2 DRGs neurons [58]. In alpha‐CGRP‐deficient mice, decreased nociception and pain behaviour were observed [66]. In OA patients, serum CGRP levels and the density of CGRP‐positive nerve fibres were highly correlated with OA‐associated pain symptoms and disease severity [21]. Studies have also shown that during angiogenesis in the arthritic joint, CGRP‐immunoreactive sensory nerve terminals grow into channels that penetrate articular cartilage [75] and along neovasculature in the inner two‐thirds of the knee meniscus [3]. With the progression of OA, therefore, joint structures that are normally not innervated might become sources of pain.

### Joint pain signalling attenuation using engineered EXOs

A truncated form of the CGRP peptide, CGRP_8–37_, is a selective antagonist that binds to the CGRP receptor with approximately the same affinity as CGRP, but does not result in receptor signal transduction (Figure 2). The analgesic effect of CGRP_8–37_ has been shown in several pain models [7, 12, 14, 16, 23, 31, 40, 51, 74, 79]. Acute blockade of joint CGRP receptors by CGRP_8–37_ normalised the enhanced mechanical evoked responses of joint nociceptors in animal models of OA, with no effects in the respective control groups [12]. We have recently generated and sequenced a DNA fragment encoding CGRP_8–37_ cDNA and have generated viral vectors (adeno‐associated virus [AAV] and lentivirus) that can be utilised in the development of gene therapeutic approaches to reduce chronic pain. The potential game‐changing benefit of targeted gene therapy is the ability to provide a local and sustained source of pain‐reducing molecules, which can be continually synthesised by the host recipient cells, reducing or eliminating the need for repeated administration. Preliminary findings in our lab have shown that direct intraspinal or intrathecal injection of AAV CGRP_8–37_ can reduce chronic neuropathic pain symptoms in rat spinal cord injury and peripheral nerve injury pain models.

AAV is widely considered the top clinically acceptable candidate due to low toxicity and the ability to provide continued transgene expression and long‐term production of desired therapeutic molecules. AAV transduction is generally a nonintegration method and therefore is considered safer than lentivirus mentioned above. There have now been over 130 clinical gene therapy trials involving AAVs, with overall good safety profile [4]. AAVs providing immunomodulatory cytokine IFNβ, TNFα soluble receptor or PD‐L1 administered via intraarticular injection have been shown in preclinical rheumatoid arthritis models to reduce hindpaw swelling, inflammation and cartilage damage [1, 2, 35, 44], and a phase 1/2 clinical trial using recombinant AAV TNF receptor showed good tolerability with modest improvement in joint swelling [55]. Emerging studies have indicated that the use of EXO‐enveloped AAVs (EXOs‐AAVs) can improve transduction, prolong transgene expression, and increase resistance to human neutralising antibodies compared with standard AAVs when administered locally to diverse sites such as intravitreal, intracochlear and intracerebral [29, 60, 80]. In addition, studies show that EXOs enhance AAV entry into cells and directly protect AAV from exogenously administered human neutralising antibody [49].

Based on this concept (Figure 2), we have recently demonstrated that engineered CD10^High^aCGRP^High^ EXOs possess a unique molecular profile, including miRNAs, lncRNAs and proteins influencing signalling pathways, suggesting analgesic and anti‐inflammatory potential in vitro. Subsequent functional and in vivo studies demonstrated that intraarticular delivery of CD10^High^aCGRP^High^ EXOs attenuated pain and preserved joint structure in OA animal models, showing anabolic effects on cartilage and identifying lncRNAs that promote cell homeostasis [46, 47].

### The evolving regulatory landscape of engineered EXOs

Engineered EXOs are generally classified primarily as biologics (or biological medicinal products), but if designed to carry functional genetic material (e.g., mRNA, siRNA, CRISPR‐Cas9), they may be categorised strictly under gene therapy or Advanced Therapy Medicinal Products (ATMPs) [9, 77]. Specifically, regulatory agencies like the Food and Drug Administration (FDA) and the European Medicines Agency (EMA) generally group EXOs (even engineered ones) as biologic drugs. This subjects them to stringent evaluation for safety, purity and potency under frameworks like Section 351 of the Public Health Service Act. However, when EXOs are intentionally engineered to harbour or express a specific sequence of nucleic acids to modify gene expression, the cargo shifts the regulatory pathway into the gene therapy domain. This means dealing with heightened scrutiny regarding the mechanism of action, off‐target effects and genetic stability.

From a scalability standpoint, as EXOs are fundamentally cellular products, manufacturing them uniformly at scale (whether through cell‐culture bioreactors or synthetic lipid bilayer assemblies) is highly difficult. This initially hinders true ‘off‐the‐shelf’ allogeneic availability compared to traditional small‐molecule drugs. Also, as the parental cell type, culture conditions, and isolation methods can alter exosome properties, maintaining identical therapeutic efficacy across different production batches remains a significant hurdle to widespread, immediate availability. However, significant industry resources are being poured into synthetic modifications to overcome these supply‐chain constraints [28, 69].

Finally, EXOs are classified as biologics, and therefore, regulators require exhaustive molecular characterisation. This demands high‐tech profiling of all membrane proteins, lipids and internal cargo to ensure batch‐to‐batch consistency. Unlike synthetic drugs, where the chemical composition is the product, the mode of action (MoA) in engineered EXOs can be highly complex, relying on the vesicle's membrane fusion capabilities, surface ligands and internal contents. Developing standardised potency assays (a requirement for Biologics License Application or marketing authorisation [MAA] approval) is one of the biggest scientific challenges holding back commercialisation [69, 77].

### Potential clinical application of analgesic engineered EXOs for OA pain

Systemic receptor antagonists for pain, including N‐methyl‐D‐aspartate (NMDA) (e.g., ketamine), CGRP (e.g., rimegepant) for migraine and opioid antagonists, are highly targeted treatments, but they frequently cause systemic adverse effects. Common side effects include nausea, dizziness, fatigue and dose‐dependent central nervous system (CNS) or gastrointestinal (GI) disturbances [15, 26, 63].

Innovative inflammatory/degenerative joint disease therapies require rethinking the overall strategy, incorporating emerging notions such as the role of synovitis/IFP fibrosis in initiating pathological events, and pro‐inflammatory resident M1 macrophages as amplifiers of the molecular cascades leading to altered joint homeostasis [37]. Both theories can be addressed through the generation and functional testing of an EXO‐based/CGRP antagonist product. Using a more homogeneous and reproducible EXO product that uniformly exhibits a specific desired attribute CD10^High^ (SP degradation and inflammation reversal) carrying pain‐reducing CGRP antagonist cargo (EXOs‐AAV CGRP_8‐37_), inherent variations in the composition of injectable therapeutics can be minimised [42] while maximising therapeutic benefit. On this basis, the proposal to utilise CD10‐enriched EXOs to enzymatically degrade SP represents a sophisticated ‘bioclearing' strategy that in combination with aCGRP cargo may offer a localised targeted therapeutic solution without the common adverse effects of systemic receptor antagonists medications.

**Figure 3:**
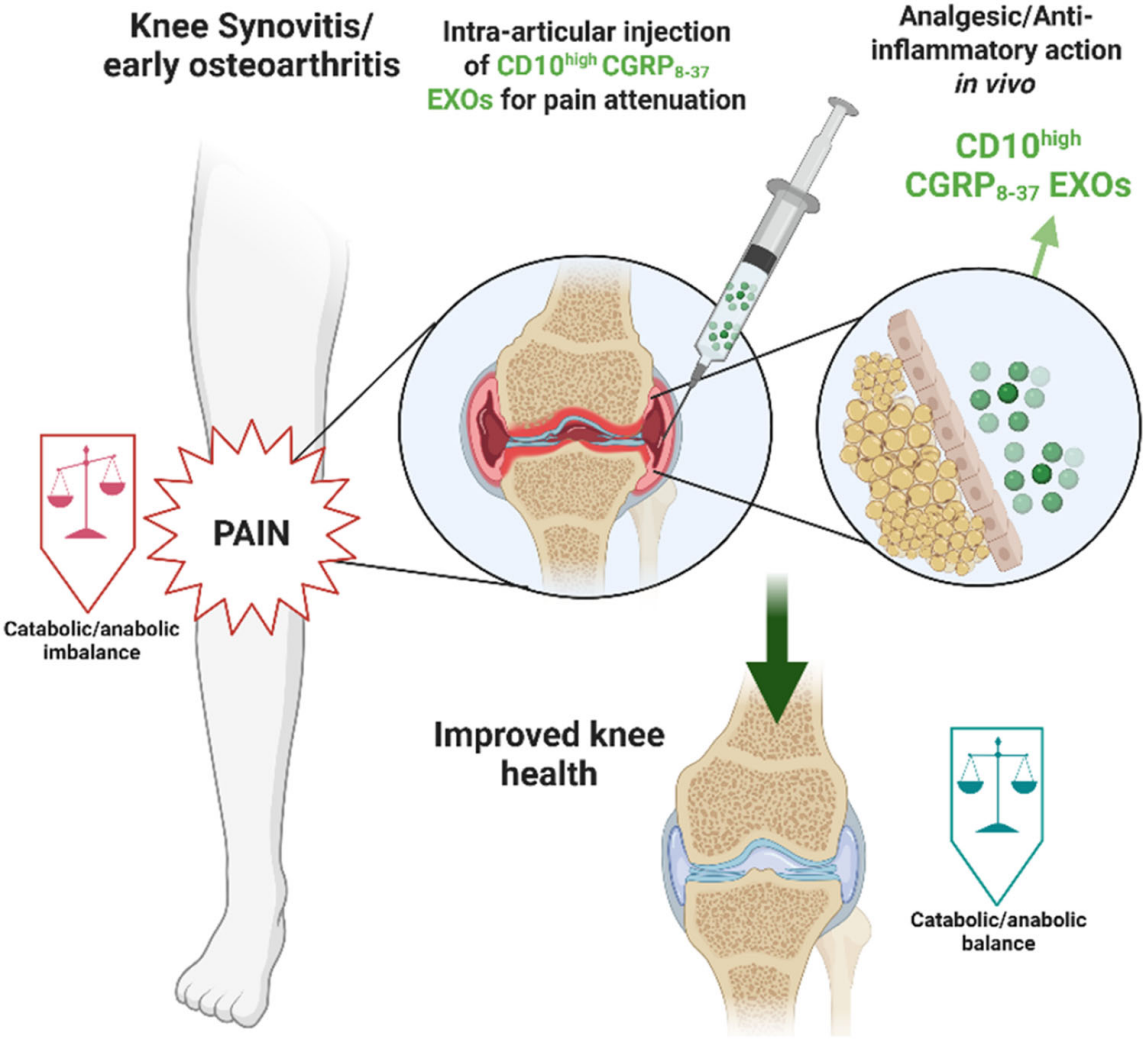
Potential clinical relevance of CD10high CGRP_8–37_ EXOs therapeutic applications. CGRP, calcitonin gene‐related peptide; EXOs, exosomes.

Human MSC cultures processing under regulatory‐compliant conditions can potentially reduce regulatory hurdles and ease ultimate clinical translation. As natural EXOs have a characteristically short retention time inside the joint capsule, therapeutic effects are often transient, requiring frequent dosing to achieve lasting analgesia. To enhance long‐term stability, researchers are developing smart‐delivery platforms (e.g., thermosensitive hydrogels or nanovesicle patches) that resist the catabolic milieu and enable the sustained, localised release of EXOs over several weeks [52]. Importantly, engineered EXOs aim to actively reprogram the inflammatory microenvironment by driving the polarisation of macrophages from an inflammatory M1 state to an anti‐inflammatory and tissue‐repairing M2 state. By altering this underlying immune imbalance, engineered EXOs can yield disease‐modifying benefits rather than just masking pain, which may ultimately extend the duration of the analgesic effect and reduce the need for constant re‐dosing. In that spirit, a standardised ‘off‐the‐shelf’ inflammation/pain‐reversing product with high reproducibility and low variability for allogeneic therapeutic schemes can hopefully evolve (Figure 3).

## CONFLICT OF INTEREST STATEMENT

The authors declare no conflicts of interest.

## ETHICS STATEMENT

The authors have nothing to report.
